# Urbanization drives phenotypic differences but not asymmetry in the damselfly *Ischnura elegans*

**DOI:** 10.1038/s41598-026-57538-7

**Published:** 2026-06-14

**Authors:** Szymon Sniegula, Guillaume Wos

**Affiliations:** Institute of Nature Conservation, Polish Academy of Sciences, Krakow, 31-120 Poland

**Keywords:** Dispersal ability, Fluctuating asymmetry, Freshwater insect, Human activities, Impervious surface, Morphology, Ecology, Ecology, Environmental sciences

## Abstract

Urbanization is a challenging environment and has a profound impact on organisms. Cities may be the source of important phenotypic changes and lead to developmental instability. To explore the effects of urbanization (percentage of impervious surface) on organisms, we collected ~ 800 adult damselflies *Ischnura elegans* across 22 ponds along an urbanization gradient. These ponds were also characterized by other environmental parameters: percentage of crops and of forested area, pH, salinity and water temperature. Adults were screened for traits related to body size and dispersion (wing morphology). We also investigated fluctuating asymmetry between right and left wings, a measure of developmental instability. We found that along an urbanization gradient, adult size and thorax length decreased, with urban individuals being smaller. The effects of urbanization were also sex-specific for femur length, with a stronger decrease in urban males. Wing shape was affected by urbanization in a sex-specific manner, with more important shape variation observed in females along the urbanization gradient. Urbanization did not increase fluctuating asymmetry, which was rather pond-specific. Our results provide evidence that urbanization shapes adult phenotype and may ultimately influence dispersal ability. Urbanization did not increase developmental instability, suggesting that some organisms may overcome some city stress.

**Supplementary Information:**

The online version contains supplementary material available at https://doi.org/10.1038/s41598-026-57538-7.

## Introduction

Human activities are causing rapid degradation of natural ecosystems and are an important cause of decline in biodiversity^1^. Urbanization has emerged as one of the main drivers of habitat transformation creating novel forms of selective pressures that may have a profound impact on organisms. Indeed, there is increasing evidence showing that urbanization may be the source of important phenotypic changes, e.g., in body size in bumblebees^2^, and evolutionary divergences, e.g., fixed faster lifestyle in urban daphnids^3^. Moreover, urban areas are generally considered a stressful environment as organisms have to adapt to many human disturbances such as artificial light, stressful temperatures and pollution^1,4^, that may have downstream effects on their development. It is predicted that stressful conditions increase development instability that may have consequences on individual fitness^5^. Hence, the effects of urbanization are manifold and to better understand its effects, it is important to investigate its impact on various aspects of organisms’ morphology and functional traits.

In urban environments, organisms experience novel or different conditions compared to non-urban environments, e.g., alternative temperatures (‘urban heat island’) or various levels of pollution^1^, with downstream effects on organisms’ life-history and morphological traits. Previous empirical studies demonstrated, for instance, a reduction in body size in urban compared with rural populations in bumblebee species^2^, or the opposite pattern in moth species^6^. Urbanization effects may also be sex-specific, with an increase in mass observed in urban males only in butterfly species^7^ and with urban males being lighter and showing decreased growth rate than rural males in the damselfly *Ischnura elegans*^8^. Some of these studies showed that differences in life history traits between urban and non-urban populations can be detected even at a microgeographic scale, i.e. separated by few dozen kilometres^6,8^, indicating that strong selection pressure in urban environments can affect local adaptation despite potential gene flows.

Moreover, urbanization may further affect traits related to dispersal, e.g., wing size and shape in insects. Urbanization causes habitat fragmentation and land use changes affecting connectivity between habitats and disrupting or favouring the dispersal of organisms through changes in wing morphology^9^. For instance, previous studies showed smaller wings in urban bumblebee species compared with rural ones inhabiting meadow or pastural land^2,10^. In addition, it was demonstrated differences in flight morphology in term of smaller thorax size (proxy for wing muscle investment) in urban compared with forested habitat^11^. These studies provided evidence that the patterns of variation in wing size and shape but also in other traits related to flight (thorax size) were dependent on the level of urbanization which may impede or facilitate insects’ movements. Hence, to have a better picture of the effects of urbanization on dispersal and flight ability, it is important to consider multiple traits related to wing morphology and flight performance.

Urbanization may also have adverse effects on organisms reflected by variation in the individual symmetry. An asymmetry between body parts generally reflects development instability and is defined as perturbations, e.g. pollution, that occur during the development that an organism is unable to buffer^12,13^. These perturbations typically disrupt homeostasis and may have effects on organism’s development and fitness^5^. A common measure of developmental instability is the measure of asymmetry between the left and right side of body parts also known as fluctuating asymmetry (FA)^12,14^. Previous studies on variation in the individual symmetry showed for instance that warmer temperature in urban areas increased wing size asymmetry in bees^10^ and head shape asymmetry in lizards^15^. However, the opposite results were also found in bees^16^ and in lizards^17^, as well as no effect of urbanization on wing symmetry in butterflies^18^. The effect of urbanization on individual symmetry is not yet clear and require further investigations.

Here, we conducted a large field sampling experiment and aimed at studying the phenotypic changes across multiple water bodies spanning an urban-rural gradient in adult damselflies. For this, we investigated the phenotypic variation related to urbanization in approx. 800 adult damselflies (males and females) sampled across 22 water bodies including ponds, lakes, and water reservoirs (“pond” hereafter). These ponds are located in Southern Poland (except for three sites located in Northern and North-western Poland), spanning different urban and non-urban environments also characterized by various levels of agricultural land, forested area in their surroundings and by different water parameters (pH, salinity and water temperature). Adults were screened for traits related to body size and wing morphology, and for wing symmetry along the urbanization gradient. In general, urban environments are associated with elevated temperatures (urban heat islands)^1^. Within the cities, ectothermic organisms commonly show faster development and growth which may be translated into smaller size^3^. Based on this, we expected a reduction in body and wing size in adult damselflies as the urbanization level increases. In damselflies, there is also a sexual size dimorphism^19^, and we expect sex-specific differences in responses to environmental conditions. Because larger female size is often correlated with increased fecundity, body size in females is likely to be more developmentally constrained and less plastic than in males. As urbanization is often considered a stressful environment, we expected higher wing asymmetry in urban habitats. We specifically asked 1) Is urbanization associated with a body size reduction in adult damselflies? 2) Do we observe increased development instability as the level of urbanization increased? 3) Are the effects of urbanization sex-specific?

## Methods

### Study species

The damselfly *I. elegans* is a common and abundant species in Europe^20^. At central latitude, including Poland, *I. elegans* has a variable voltinism and completes one (univoltine cohort) or two generations (bivoltine cohort) per year^21^. Comparing to relatively brief adult stage, *I. elegans* have a much longer aquatic larval stage, typically lasting from several months up to one year. In a univoltine cohort, larva is the overwintering stage. The species has asynchronous emergence and breeding phenology with adults emerging and breeding in spring and summer^22^. After egg laying, hatching takes place between two-three weeks after.

### Collection sites and dates

**Fig. 1:**
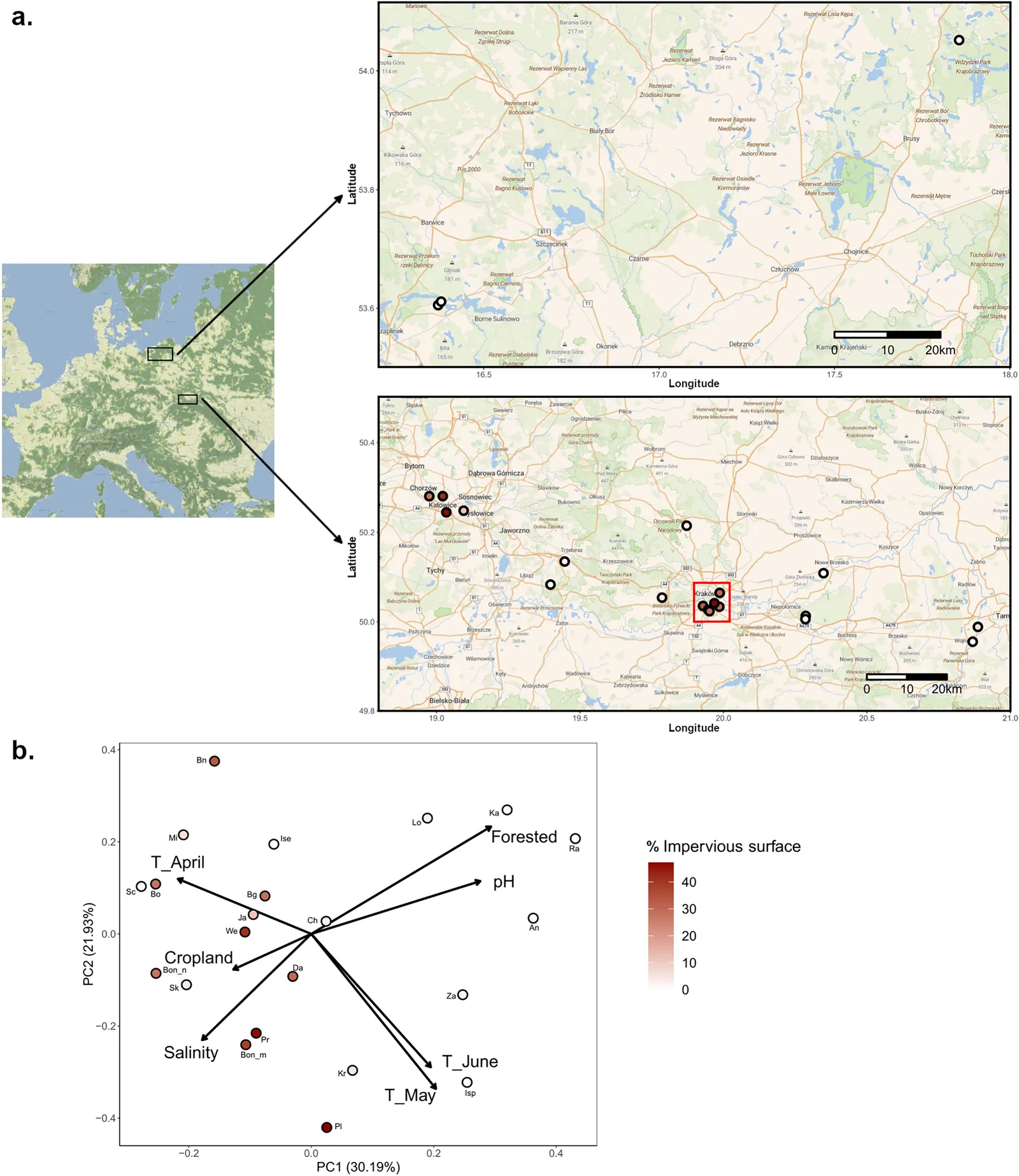
**a**) Locations of the 22 ponds in Southern and Northern Poland (© Stadia Maps © OpenMapTiles © OpenStreetMap; https://stadiamaps.com/, https://openmaptiles.org/, https://www.openstreetmap.org/copyright; R package ‘ggmap’^67^). An enlarged map of the ponds located in the red square is available in Fig. S1. **b**) Principal component analysis of the seven environmental parameters measured on the 22 ponds: Salinity, pH, percentage of cropland (cropland) and of forested habitat (forested), water temperature for the month April (T_April), May (T_May) and June (T_June). Ponds are coloured based on the percentage of impervious surface (Table S1).

We sampled adult *I. elegans* using insect sweep nets from 22 ponds during the first half of the flying season (end of May to mid-July 2024) so that sampled adults belong to same cohort, thereby reducing temporal variations between populations^21^: 19 ponds are located in southern Poland and three ponds are located in northern Poland (Fig. 1a; Fig. S1). In addition, for six ponds, we also included adult samples from previous studies collected in 2020^23^, 2021^8,24^, 2022 (unpublished data) and 2023 (unpublished data) during the first half of the flying season (Table S1). In total, 825 adults were collected across all ponds and years (number of sampled adults per pond range from 16 to 57; median = 33 with sexes pooled), including 421 females and 404 males. The detailed sample size is included in Table S1.

For each pond, the urbanization level was quantified using the percentage of impervious surface, a common measure used to assess the effects of urbanization^25,26^. For this, we created a circular buffer of 1 km diameter around each sampling site, the centre of each circle was placed on the shore of each pond where the sampling was made, and calculated the average value of urbanization in each buffer. We also measured five other parameters: percentage of cropland and of forested habitat surrounding the sampling site using a similar method as for the urbanization level, pH and salinity (CPC-105, Elmetron, Poland) and water temperature (“temperature” hereafter with dataloggers HOBO UA-001-64, Onset, Fig. S3), (Table S1). We estimated the percentage of cropland as a proxy of agricultural pressure and of forested habitat because it may impact dispersion^27^. We measured water temperature because it is an important determinant of growth and development in aquatic ectotherms. Water temperature was measured for the month of April, May and June that correspond to the main larval growth period. Salinity and pH are linked with industrial and agricultural activities and are known to impact freshwater organisms^28,29^. Detailed descriptions and justifications for the use of these characteristics are described in File S1.

### Traits measured in adults

For all adults collected, we measured three morphological traits from pictures using a microscope (Nikon SMZ745T) mounted with a digital camera (The imaging source DFK 23UP031; zoom x10, distance to objects = 12 cm): head width (a proxy for overall body size in damselflies^19^), femur length (linked to body size^30^ and prey capture ability^31^), and thorax length (associated with flight muscle investment and flight capacity^31^) (Table S1). The three variables were not redundant and had correlation coefficients below 0.7 (Table S2).

**Fig. 2:**
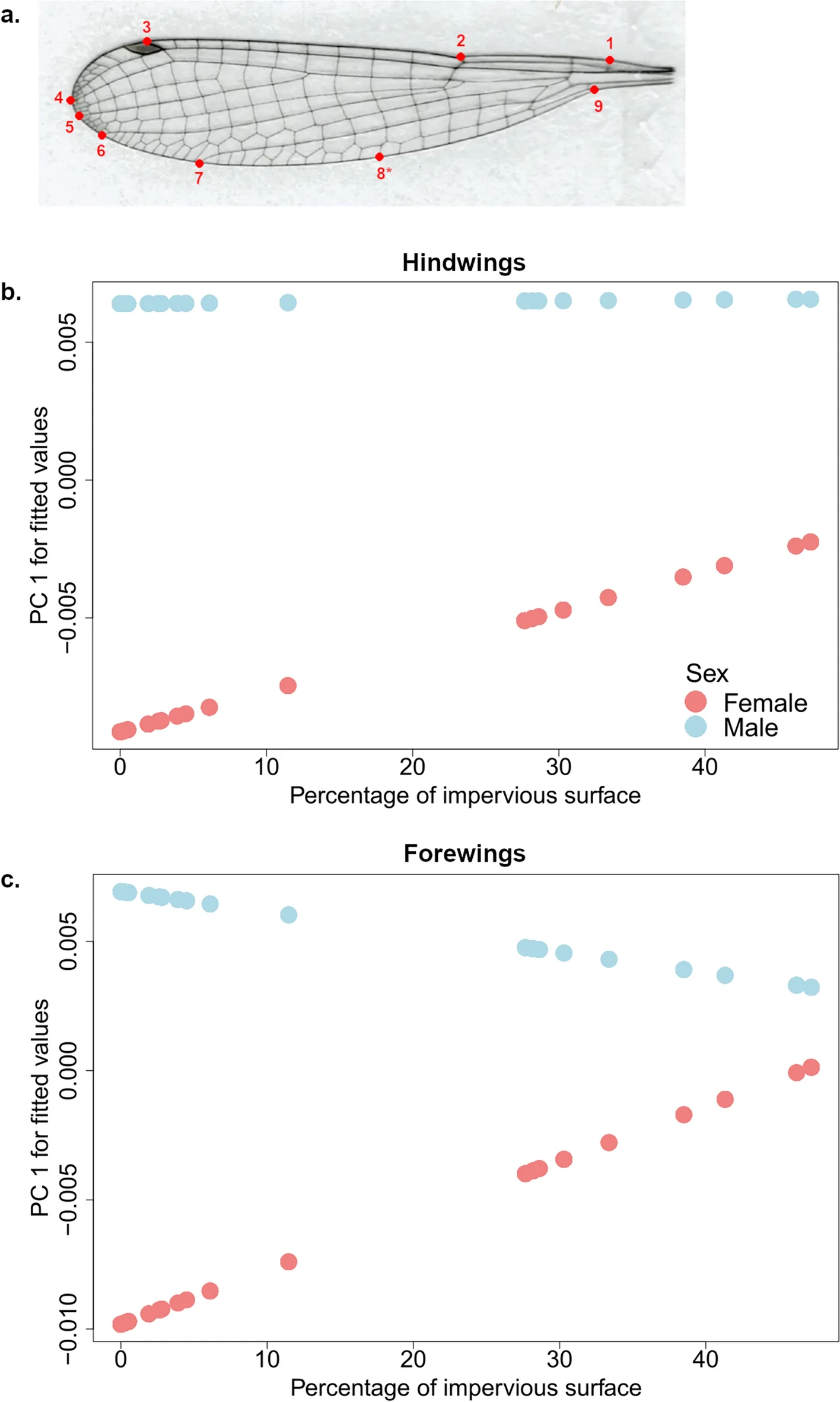
Wing shape analysis showing in **a**) the position of the nine landmarks defining the outline of a wing. The asterisk indicates the semi-landmark. In *I. elegans*, the hind- and forewings are very similar and have the same veins, the position of the landmarks were the same for both wings. Allometry plots showing variation of the first principal component of the fitted values (predicted shapes) for the **b**) hindwings and **c**) forewings along the percentage of impervious surface (proxy of urbanization) for males and females. The slopes of each line represent the rate of shape change relative to a change in the percentage of impervious surface. Dots indicate ponds and are coloured by sex.

In addition, we collected the hind- and forewings for a wing morphology analysis to estimate wing size and shape, two parameters linked with dispersal and flight ability. Damselflies have one pair of hindwings (left and right) and one pair of forewings (left and right) totalling four wings per individuals. For the wing morphology analysis, we adapted the procedure described in^32^. Individuals with all wings damaged were discarded. For some individuals, only wings from the left or the right side were damaged. The highest sample size was obtained when using only the left hind- and forewings, consequently, we performed the analysis of wing shape and size using only the wings from the left side. The wing morphology analysis was done on 797 adults. For each adult damselfly, we prepared the wings as follow: for each individual, wings were stuck on a sheet of paper in order to be digitized. Next, we placed eight landmarks and one semi-landmark along the wing outline using tpsDig2^33^ (Fig. 2a). The landmarks are fixed points while the semi-landmark is allowed to slide along the outline to minimize the Procrustes distance between each wing and the average configuration. The same landmarks were used for the hind- and forewings as they have similar venation patterns. Next, we ran a generalized Procrustes analysis to remove the effects of position across wing samples and ensure compatibility across all samples (‘gpagen’ function, package geomorph^34^). After the alignment of wing samples, wing coordinates were used to estimate wing size (Table S1) and shape and for the bilateral symmetry analysis.

### Fluctuating (FA) and directional (DA) asymmetry analysis

Fluctuating asymmetry (FA) reflects deviation from normal symmetry and is due to random perturbations that occurs during the development and is relevant to estimate the impact of environmental stressors on organisms^14^. Hence, FA corresponds to developmental noise and needs to be distinguished from directional asymmetry (DA) which reflects consistent, non-random deviation from normal symmetry within a population and may reflect specific adaptations or specific functions. We assessed FA on wings as this trait is commonly used for symmetry analysis in damselflies and higher symmetry is positively linked with reproductive success^35,36^.

For the symmetry analysis, wings were prepared as described above, and wing coordinates were used as input files. We used both the left and right hind- and forewings. The sample size was lightly smaller due to the lower number of individuals with all their wings intact. Across the 22 ponds, we collected a total of 353 pairs of hindwings for males and 363 for females and 353 pairs forewings for males and 361 for females. Detailed sample size per pond is provided in Table S1.

First, we tested for the effects of FA and DA on the shape of damselfly wings using bilateral symmetry analysis (‘bilat.symmetry’ function, package geomorph^31^). The function estimates the deviation in shape between the right and left wing. For the bilateral symmetry analysis, we ran separate models for each pond, hind- and forewings, and males and females. A deviation in shape symmetry between the right and left wing that is individual-specific within a pond indicates FA. At the opposite, a consistent deviation in shape symmetry between the right and left wing shared across all individuals of the same pond reflects DA. Significance of FA and DA on wing shape was tested by ANOVA implemented in the bilat.symmetry function. To take into account measurement errors, the positioning of landmarks on each hind- and forewing were performed twice^37^ and replicated measurements were included in the bilat.symmetry function.

As we detected significant level of FA in each pond, we further tested whether FA was related to urbanization. For this, we extracted from the ‘bilat.symmetry’ function the unsigned asymmetry index (unsigned.AI) in order to assign to each individual a unique asymmetry value defined as the Procrustes distance (shape deviation value) between the right and left wing that was used as a proxy for FA^38,39^.

### Spatial autocorrelation

For the traits measured in adults: femur length, thorax length, head width, wing size and unsigned.AI, we tested for spatial autocorrelation using the function ‘testSpatialAutocorrelation’ (package dharma^40^) based on Moran’s I test. Spatial autocorrelation was not significant for femur length (Moran I = 0.120, p-value = 0.263), thorax length (Moran I = 0.109, p-value = 0.298) head width (Moran I = 0.225, p-value = 0.067) and FA (Hindwings: Moran I = -0.093, p-value = 0.757, forewings: Moran I = -0.098, p-value = 0.732) but was significant for wing size (Moran I = 0.350, p-value = 0.008). Spatial autocorrelation was not tested for wing shape because the function used to analyse shape data (procD.lm implemented in geomorph package^31^) was not supported. As spatial autocorrelation was significant for wing size, we supposed that it was also significant for wing shape. To consider spatial autocorrelation for the analysis of wing size and shape, we added latitude and longitude of each pond as covariable in the model, after adding these two covariables, spatial autocorrelation was no longer significant for wing size (Moran I = -0.334, p-value = 0.9256).

### Statistical analyses

All statistical analyses were performed in R^41^. First, we ran a principal component analysis on the 22 ponds sampled including all the pond characteristics: urbanization (= percentage of impervious surface), temperature (for April, May and June), percentage of cropland and forested habitat, pH and salinity. All variables were scaled and centred.

We also performed a correlation analysis between all the pond characteristics using Spearman rank correlation and between the phenotypic traits (femur length, thorax length, head width and wing size) using Pearson correlation coefficients. P-values were corrected for multitesting using Bonferroni correction by dividing the alpha level by the number of tests (α/8 for the environmental parameters and α/5 for the morphological traits).

For the adult traits: femur length, thorax length, head width and wing size we ran generalized linear model (glmmTMB package^42^), with sex, urbanization (as a continuous variable), and their interaction included as fixed effects; pond and year of collection (year) nested within pond were included as random factors. The four variables followed a normal distribution. For wing size only, we included latitude and longitude as covariates to account for spatial autocorrelation, and head width as another covariate to account for body size because these two variables strongly correlated. For unsigned.AI, we ran two separate generalized linear models for the hind- and forewings with unsigned.AI as a response variable; unsigned.AI was log-transformed. The models included the two-way interactions between sex and urbanization (as a continuous variable) as fixed effects; pond and year nested within pond were added as random factors. P-values were obtained using the Wald chi-square test (Chisq) implemented in the car package^43^. Significance and percentage of the variance explained by the random factor was tested using lmtest (lmtest package^44^) and intraclass correlation coefficient (performance package^45^). For each significant variable, we estimated the effect size using partial eta-squared (η2) using ‘etaSquared’ function (package lsr^46^) with values of 0.01 and below indicating small effects, of 0.06 indicating medium effects and above 0.14 indicating large effects. We also calculated 95% confidence intervals using ‘confint’ function in R.

For wing shape, the model was fitted using the function ‘procD.lm’ implemented in the geomorph package^34^. We used sex, urbanization (as a continuous variable) and their interaction as fixed effects, and added latitude, longitude and head width as covariates. Pond and year nested within pond were used as random factors. For the ‘procD.lm’ function, p-values were computed using ANOVA and effect sizes (z scores) were calculated based on cohen’s f^2^ (analogous to partial eta-squared) in order to provide information on the magnitude of the significant effects. Values were log-transformed before z-score calculation.

## Results

### Environmental variation across the 22 ponds

Principal component analysis showed that along the urbanization gradient depicted by the percentage of impervious surface (PC1, 30.19%), the 22 ponds mostly separated according to their values of salinity and percentage of forested habitat (Fig. 1b, Table S2), with higher salinity, and lower percentage of forested habitat as urbanization increased. Temperature in April, pH and percentage of crops also varied along PC1 but were not significantly associated with urbanization. Along the second axis (PC2, 21.93%), ponds separated mostly based on their water temperature especially in May and June, however, the direction did not seem to be linked with urbanization but seemed rather to be pond-specific.

### Adult morphology

**Fig. 3:**
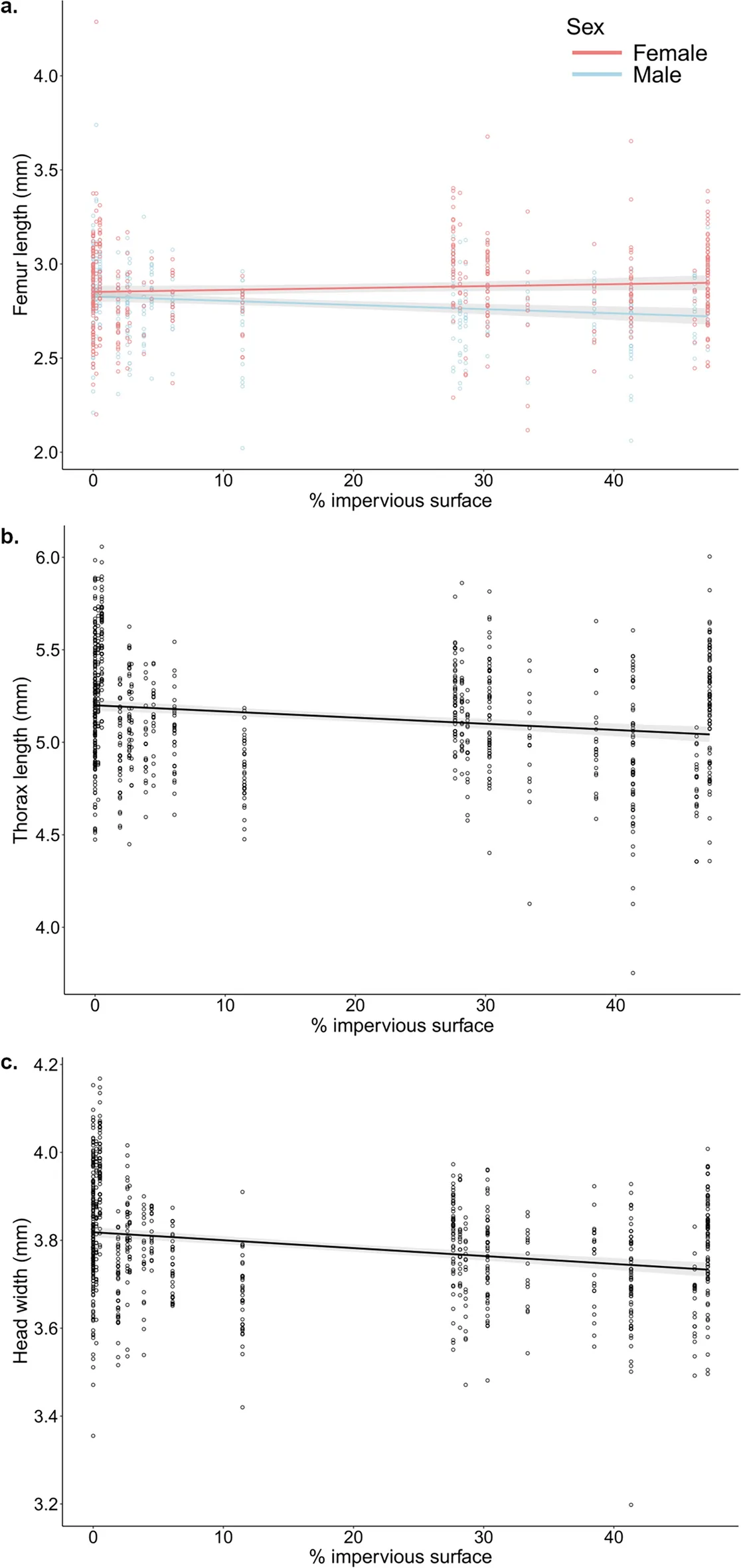
Linear regressions showing **a**) the interactions between sex and urbanization (% of impervious surface) for femur length and the effects of urbanization for **b**) thorax length and **c**) head width. Females are coloured in red and males in blue. Each dot depicts one individual. Grey area represents standard error.

The analysis of the three phenotypic traits: femur length, thorax length and head with, showed different patterns (Table 1, Fig. 3). For femur length, we found significant effects of sex (Partial eta-squared [η2] = 0.03, confidence intervals [CI] = -0.06;-0.02) with longer femur in females. The effects of urbanization were sex-specific (η2 = 0.02, CI = -0.005;-0.002) values for femur length decreased for males along the urbanization gradient and no detectable effect was observed for females (Fig. 3a). For thorax length, we found significant effects of sex (η2 = 0.17, CI = -0.23;-0.13) with shorter thorax in males. For thorax length and head width, we found significant effects of urbanization. Values for thorax length (η2 = 0.07, CI = -0.004;-0.001) and head width (η2 = 0.07, CI = -0.002;-0.000) decreased with a similar magnitude as the percentage of impervious surface increased (Fig. 3b, c). We noted that for thorax length, the interaction sex × urbanization was marginally significant and, as a trend, followed the same pattern as for femur length. For all traits, we also found significant effects of the random factors pond and pond:year explaining 13.5 % and 14.7% of the variance in femur length, 26 % and 29.7% in thorax length and 27.5 % and 31.5% in head width respectively.

**Table 1:** Results of the generalized linear models showing the effects of sex, the percentage of impervious surface (urbanization), and the interaction between urbanization and sex on the three morphological traits (head width, femur length and thorax length). The effects of the random factors pond and pond:year are also shown.

		Femur length	Thorax length	Head width
Variables	Df	*p* (Chisq)	*p* (Chisq)	*p* (Chisq)
Sex	1	< 0.001 *** **(14.0)**	< 0.001 *** **(158)**	0.784 (0.07)
Urbanization	1	0.599 (0.49)	0.035 * **(4.43)**	0.023 * **(5.20)**
Urbanization × Sex	1	0.021 * **(5.30)**	0.085 (*) (2.97)	0.398 (0.71)
Random effects	Df	*p* (Chisq)	*p* (Chisq)	*p* (Chisq)
Pond	1	< 0.001 *** **(81.1)**	< 0.001 *** **(284)**	< 0.001 *** **(270)**
Pond:year	1	< 0.001 *** **(76.3)**	< 0.001 *** **(307)**	< 0.001 *** **(286)**

Table shows degrees of freedom (Df), p-values and Wald Chi-squared test in parentheses. Significance is indicated in bold: *** p < 0.001, * p < 0.05, (*) p < 0.1.

### Wing morphology

The analysis of wing morphology revealed that the size of the hind- and forewings was not affected by urbanization (Table 2). For the hindwings, sex had the largest effects on wing size with females having bigger wings than males (η2 = 0.69, CI = -0.22;-0.20). Then head width had the second largest effects (η2 = 0.27, CI = 0.35;0.44) with bigger wings in individuals with larger head width, followed by latitude (η2 = 0.08, CI = 0.03;0.05), with bigger wings at higher latitudes in the three ponds from northern Poland. The random factors pond and pond:year were also significant and explained 3.5 % and 5 % of the variance respectively. For the forewings, we also found effects of sex (η2 = 0.70, CI = -0.24;-0.21), head width (η2 = 0.28, CI = 0.39;0.48) and of latitude (η2 = 0.08, CI = 0.03;0.05) of similar magnitude and going in similar directions as for the hindwings. The random factors pond and pond:year were also significant and explained 2.9 % and 4 % of the variance respectively.

**Table 2:** Effects of urbanization, sex and their interaction on wing size and shape for the hind- and forewings. Adult head width (proxy of body size), latitude and longitude were added as covariates and pond and pond:year as random factors. For wing size, table shows results of generalized linear model. For wing shape, table shows results of the procD.lm function.

		Hindwings		Forewings	
		Shape	Size	Shape	Size
Variables	Df	*p* (*z*)	*p* (chisq)	*p* (*z*)	*p* (chisq)
Head width	1	0.05(*) (1.66)	< 0.001 *** **(193)**	0.045* **(1.72)**	< 0.001 *** **(202)**
Latitude	1	< 0.001 *** **(4.73)**	< 0.001 *** **(13.6)**	< 0.001 *** **(5.54)**	< 0.001 *** **(16.1)**
Longitude	1	0.558 (-0.15)	0.079 (3.09) (*)	0.349 (0.39)	0.107 (2.60)
Sex	1	< 0.001 *** **(5.81)**	< 0.001 *** **(1622)**	< 0.001 *** **(5.62)**	< 0.001 *** **(1686)**
Urbanization	1	0.327 (0.46)	0.226 (1.47)	0.175 (0.95)	0.435 (0.61)
Urbanization × Sex	1	0.012 * **(2.26)**	0.135 (2.21)	< 0.001 *** **(4.29)**	0.281 (1.16)
Random effects	Df	*p* (*z*)	*p* (chisq)	*p* (*z*)	*p* (chisq)
Pond	18 (shape)/1 (size)	< 0.001 *** **(2.94)**	< 0.001 *** **(69.6)**	< 0.001 *** **(5.52)**	< 0.001 *** **(63.8)**
Pond:year	9 (shape)/1 (size)	< 0.001 *** **(6.00)**	< 0.001 *** **(91.6)**	< 0.001 *** **(5.95)**	< 0.001 *** **(73.4)**

Table shows degrees of freedom (Df), p-values and Wald Chi-squared test (chisq, in parentheses) for wing size, and p-values and z scores (effect size based on cohen f^2^ distribution, in parentheses) for wing shape. Significance is indicated in bold: *** p < 0.001, * p < 0.05, (*) p < 0.1.

For wing shape, we found significant effects of latitude, sex and year for both the hind- and forewings. The effects of the variables were of similar magnitude between the hind- and forewings. For both types of wings, we found a significant interaction sex × urbanization. The effect of the interaction was more pronounced on the forewings (effect size, z score = 4.30) compared with the hindwings (z score = 2.26). To separate the effects of sex, we computed the z scores for the effects of urbanization for males and females separately. For the hindwings, the effect size of urbanization on wing shape was 0.25 for males and 0.91 for females. For the forewings, the effect size of urbanization was -0.37 for males and 2.11 for females. Males and females had different allometry trajectories and the rate of wing shape change along the urbanization gradient was higher for females (steeper slope) than for males for both the hindwings and forewings (Fig. 2b and c). Along the urbanization gradient, wing shape of females varied mostly on the shape of the trailing edge of the wing (Fig. S4 and Fig. S5). For wing shape, the effect of the random factors pond and pond:year were also significant and explained 3.4 % and 7.8 % of the variance for hindwings and 5 % and 6.4 % for forewings respectively.

### Developmental instability

We tested for the effects of fluctuating asymmetry (FA; developmental noise) and directional asymmetry (DA; non-random deviation) on damselfly wings across the 22 ponds, for males and females, and for the hind- and forewings (Table S3). The hind- and forewings of damselflies were significantly affected by FA in all tests across all ponds and sexes. For the hindwings, the difference in shape variation between the left and right wings explained by FA is on average 17.3 % ± 4.2 % for males and 19.3 % ± 9.2 % for females. For the forewings, the variance explained by FA is on average 19.9 % ± 4.6 % for males and 20.5 % ± 5.0 % for females. The wings of damselflies were not affected by DA except for few ponds for the forewings (in females from Lowiecki, Rakowskie and Isep and in males from Schodno) and hindwings (in females from Ispina). The percentage of variance explained by measurement errors, was generally low and ranged between 3 and 8 %, except for four tests (between 11 and 16 %), but was always lower that the percentage of variance explained by FA. Fluctuating asymmetry between the right and left pair of wings is similar for the hind- and forewings and the differences, in term of variation between individuals, are mostly on the shape of the trailing edge of the wing (Fig. S6).

As we detected significant level of FA in our dataset, we further tested whether FA was affected by urbanization using the unsigned.AI as a proxy of FA (Table 3). We did not find significant effects of sex, urbanization and of their interaction on FA indicating that the shape of the left wing was not significantly different than the shape of the right wing along the urbanization gradient. But we found significant effects of the random factors pond and pond:year on FA which explained 5.2 % and 4.3 % of variance for the hindwings and 10.2 % and 8.9 % for the forewings respectively.

**Table 3:** Effects of urbanization, sex and their interaction on unsigned.AI (fluctuating asymmetry index) for the hind- and forewings. Pond and pond:year were added as random factors.

		Hindwings	Forewings
		Unsigned.AI	Unsigned.AI
Variables	Df	*p* (Chisq)	*p* (Chisq)
Sex	1	0.463 (0.54)	0.330 (0.95)
Urbanization	1	0.498 (0.46)	0.371 (0.80)
Urbanization × Sex	1	0.359 (0.84)	0.503 (0.45)
Random effects	Df	*p* (Chisq)	*p* (Chisq)
Pond	1	0.007 ** **(7.11)**	< 0.001 *** **(17.8)**
Pond:year	1	0.019 * **(5.44)**	< 0.001 *** **(20.0)**

Table shows degrees of freedom (Df), p-values and Wald Chi-squared test in parentheses. Significance is indicated in bold: *** p < 0.001, ** p < 0.01, * p < 0.05

## Discussion

In this study, we aimed at identifying phenotypic variation associated with urbanization, defined as the percentage of impervious surface, in 22 ponds. Overall, our results confirmed the impact of urbanization on traits related to body size, flight performance and prey catching, with a general decrease in these variables when urbanization level increased. We also demonstrated that urbanization affected wing shape, yet in a sex-specific manner. Urbanization did not increase fluctuating asymmetry, suggesting that, in our study, urbanization did not induce strong developmental perturbations during the aquatic larval stage that carry-over to the adult stage. Finally, we found pond-specific and temporal effects on analysed variables, suggesting that unmeasured heterogeneity of each pond and interannual variation may shape trait expression in more complex ways. These findings indicate that while urbanization is an important ecological factor affecting damselfly phenotype, other environmental variables may also contribute to morphological variation.

### Effects of the urban environment on adult body size

In urban environments, temperature is generally higher compared to non-urban environments^1^. After compiling temperature data for our 22 ponds, we found that water temperature was not consistently higher as urbanization increases. This may be explained by the fact that water temperature may depend on numerous parameters such as the time of the year, water depth, surface area, water movement (wind and currents), and water transparency that were not assessed here. In addition, a part of our temperature measurements was taken in a single year which limits insight into year-to-year environmental variations. Urbanization correlated negatively with the percentage of forested habitat and positively with salinity. The percentage of forested habitat may affect wing size as demonstrated in the damselfly *Calopteryx maculata* with shorter wings in forested compared with pastural habitat^27^. For salinity, the observed pattern is consistent with previous reports that salinization of freshwater ecosystem is tightly linked with human activities e.g., mining, agriculture, road salt^28^. A previous study on *I. heterosticta* showed a tolerance level to salinity up to 20 mS.cm^-1^^47^. In our study, salinity is probably within the range of tolerance in *I. elegans* as the highest salinity measured was 0.768 g.L^-1^ (Bonarka M pond, corresponding to a conductivity of 1.507 mS.cm^-1^, Table S1). Noteworthy, the effects of salinity may also be dependent on other environmental factors such as temperature that may influence osmoregulation, increase ion absorption and exacerbate the effects of salinity^48^. Hence, we cannot exclude potential interactive effects between water parameters.

We found overall reduction in head width, thorax length and femur length along the urbanization gradient. The reduction in body size was consistent with previous studies in bumblebee species^2^ and in the spider *Araneus diadematus*^49^. Smaller thorax length is related to reduced investment in flight muscle but, in our study, decreased thorax length did not come with a reduction of wing size. This result contrasted with a previous study in *Triatoma dimidiata* where smaller thorax and wings were observed in individuals from urban areas^11^. For femur length, the effects of urbanization were sex-specific with a decrease in length as urbanization increase observed in males, indicating that some traits related to body size or behavior in catching prey may vary with urbanization in a sex-specific manner. Such an interaction between sex and urbanization on morphological traits was already reported in a butterfly species, with heavier adults in urban areas^7^ and in *I. elegans*, with smaller final instar-larvae before emergence in urban areas^8^ , the latter representing a proxy of adult size at emergence, which is fixed at metamorphosis^50^. In damselflies, there is a strong sexual dimorphism with females being larger than males and showing different life-history and behavioural strategies^19^. For males, development time is important to emerge before females to ensure reproduction and males are generally more plastic than females for mass- and size-related traits^8^. For females, maintaining a high body mass, and hence larger body size, is important for reproduction and this may constrain their plasticity for this trait. We may hypothesize that females may also have higher food requirement than males that may affect their predation behaviour but this would require further examinations.

In general, the effects of urbanization on organisms’ phenotype are attributed to higher temperatures in urban environments, which did not seem to be the case in our study. Therefore, the effects of urbanization on phenotype cannot be solely explained by an effect of temperature. However, other variables may also vary along the urbanization gradient such as lower food availability, pollutants or other selective pressures inducing stressful conditions that may accelerate or slow down development, e.g. predation pressure^51^, which were not quantified here.

### Effects of the urban environment on wing morphology

We further explored variation in wing shape and size used here as a proxy to estimate dispersion ability. Wing shape and size are associated with variation in wing loading, aerodynamism, flight ability and behaviour^52^ and maybe linked to fitness^53^. For wing size, despite showing significant intraspecific variation caused by latitude, sex and collection year, our results did not show an effect of urbanization. This result contrasted with previous studies^2,10^. However, other landscape characteristics may correlate with wing size such as forested habitats^27^. In our study, urbanization was negatively correlated with the percentage of forested habitats and some of our rural ponds are located inside or close to forests that may create ecological barriers similar to urban structures that may affect traits related to dispersion^27^. Moreover, the patterns of wing size variation may also be driven by the biology of the study system, e.g., wing size variation dependent on foraging behaviour in bumblebees^54^, making the underlying mechanisms of wing size variation complex.

The effects of urbanization were observed only on wing shape and were also sex-specific. Variations in wing shape, even at a microgeographic scale, were already reported in the butterfly *Pararge aegeria*^55^ and in the damselfly *C. maculata*^27^. But, no variation in wing size and shape between urban and rural populations, was found in a previous study in *I. elegans*, despite a significant variation between males and females^56^. This may be explained by the biology of the species and may depend on the dispersion potential. In *I. elegans*, the dispersal ability is pond-specific and dependent on landscape characteristics and seems to be greater in open habitats^57^ and may also be sex-specific with, as a trend, females dispersing farther than males^58^. The effect of urbanization on wing morphology seemed to be rather limited to some wing characteristics as shown in our current study and in a previous one in the damselfly *Coenagrion puella*^59^. Furthermore, wing shape has been shown to vary in response to environmental stressors such as temperature, e.g. in moths^60^, suggesting that wing shape may be a plastic trait. However, it is difficult to assess to what extent the variation in shape affect wing loading and flight ability without further kinematic investigations. It is generally assumed that such variations in wing morphology ultimately affect flight behaviour which is supposed to be adaptive^52^.

### Effects of the urban environment on fluctuating asymmetry

Fluctuating asymmetry is generally due to perturbations that occur during the development and these perturbations are mostly driven by the environment but can also be genetically-induced^12,13^. Hence, FA can also be used as an indicator of environmental disturbance and stress. For example, pollution is known to increase FA^61^, and this, in contrast to DA, which reflects specific adaptations. In our study, we did not find strong evidence for DA, but we found significant FA in wings in all ponds studied. However, contrary to our expectations, urbanization had no effects on FA. Instead, variations in FA were pond- and year-specific. This means that the level of stress sufficient to induce FA may depend on other parameters linked with agricultural practice, pond management or other sources of contamination. For instance, the presence of pesticides linked with agricultural practice may increase FA in the damselfly *Xanthocnemis zealandica*^62^ or that FA was correlated with heavy metals in the brown trout *Salmo trutta fario*^63^. This would require a thorough analysis of water parameters to clearly identify the cause of higher FA in some ponds. Furthermore, a study conducted on the damselfly *C. puella* showed that other factors such as intraspecific competition and female availability may influence FA^64^, suggesting that the patterns of FA may be influenced by a combination of abiotic and biotic factors.

Finally, we demonstrated that the percentage of impervious surface significantly affected various aspects of the phenotype of adult damselflies over a relatively short geographic scale. In addition, unmeasured pond heterogeneity and interannual variation also participated in phenotypic variation even between ponds separated by few hundred metres or few kilometres. Most of the phenotypic variation observed in adults is probably attributed to phenotypic plasticity. Indeed, a previous study found no clear genetic differentiation between ponds located in Southern Poland (including some of our sampled ponds) suggesting a strong gene flow at a local scale^65^. However, we cannot completely rule out some genetic differences even between geographically close water bodies as selection is more likely to act on few genes of large effects rather than on a large fraction of the genome^66^. Supporting this, a previous study found phenotypic divergence in life-history traits between two local populations of *I. elegans* separated by only a few kilometres^23^. These differences persisted under common garden rearing, indicating a likely genetic basis even at a microgeographic scale. Overall, the relative genetic homogeneity in our study system would also decrease the importance of a genetic cause of the increased FA in some of our ponds, which was more likely due to environmental quality rather than genetic isolation.

## Conclusions

Here, we studied the effects of urbanization on the phenotype of adult damselflies and our key findings were that: 1) the decrease in body size along the urbanization gradient was partly sex-specific and highlight the importance of considering both sexes in species with strong sexual dimorphism. 2) Urban habitats affected wing shape in a sex-specific manner and may impact the dispersal ability and flying behaviour that may ultimately affect population dynamics. 3) Urbanization did not increase FA suggesting that, to some extent, some species may overcome city stress. However, city stress may also be manifested in other ways e.g., trade-offs between metabolic functions.

## Supplementary Information


Supplementary Information 1.
Supplementary Information 2.
Supplementary Information 3.


## Data Availability

Data supporting the study are available in supplementary information (Table S1).
