# Supplementary material for: Urbanization drives phenotypic differences but not asymmetry in the damselfly Ischnura elegans
Source: Sci Rep. 2026 Jun 14;16:27142. doi: 10.1038/s41598-026-57538-7 (PMC13526801; doi:10.1038/s41598-026-57538-7)
Supplement: Supplementary file 1 [file 41598_2026_57538_MOESM1_ESM.docx]

**Supplementary information**

**Urbanization drives phenotypic differences but not asymmetry in the damselfly *Ischnura elegans***

Szymon Sniegula^1^, Guillaume Wos^1,^*

^1^Institute of Nature Conservation, Polish Academy of Sciences, 31-120 Krakow, Poland

* Author for correspondence:

Guillaume Wos

[wos.gui@gmail.com](mailto:wos.gui@gmail.com)

**File S1.** *Collection sites – Environmental characteristics*

For each pond, we measured four water parameters: pH, conductivity (µS.cm^-1^), salinity (g.L^-1^) using a conductivity meter (CPC-105, Elmetron, Poland) and water temperature (dataloggers HOBO UA-001-64, Onset) as our study species develops and grows during aquatic larval stage. The first three parameters are linked with industrial and agricultural activities and known to impact freshwater ecosystems and to increase with urbanization level ^1,2^. Similarly, it is generally assumed that water temperature increases along the urbanization gradient due to the ‘urban heat island’ effect which affects growth and development of ectotherms. We measured pH, conductivity and salinity in July and August 2024 and we took two measures per pond at different locations; we then took the mean across the two values. For eight ponds, water parameters were also measured in 2025 to show that these values are consistent from one year to another as no significant differences was found in pH (*F* = 0.45, *p* = 0.511), conductivity (*F* = 0.06, *p* = 0.816) and salinity (*F* = 0.00, *p* = 0.929) between 2024 and 2025 (Table S1). For one sampling site: Schodno, we were not able to measure these parameters. We retrieved pH and conductivity values from previous research reports available for the year 2019 and 2022 (https://www.gov.pl/web/gios/program-badan). These measures were 7.1 and 6.8 for pH and 721 and 731 µS.cm^-1^ for conductivity, respectively for 2019 and 2022; we took the mean across the two values. For Schodno, we estimated the value for salinity based on a regression line between conductivity and salinity measured for the 21 other ponds as these two parameters strongly correlate (R = 1.0, p < 0.001, Fig. S2). Because of the strong correlation between salinity and conductivity, we kept only salinity for the subsequent analysis. We recorded water temperature for April, May and June in each pond which corresponds to the main period of larval development as the peak of the flying season is in June/July. Dataloggers were placed at approx. 30 cm depth. For most of the ponds, temperature was measured in 2025, for others it was measured between 2021 and 2024. For 2 sampling sites: Schodno and Kryspinow, we were not able to place dataloggers, temperature was estimated using lake model (Flake; Mironov 2008) (Fig. S3).

We quantified the percentage of impervious surface (proxy of urbanization), of periodically herbaceous areas (proxy of cropland) and of forested habitat from the high-resolution layer database ^4^ in Quantum-GIS ^5^. We included the percentage of cropland to reflect agricultural pressure and of forested habitat to reflect natural barrier than may affect species dispersion ^6^. For this, we created a circular buffer of 1 km diameter around each sampling site, the centre of each circle was placed on the shore of each pond where the sampling was made, and calculated the average value of urbanization, cropland and forested area in each buffer (Table S1). Spearman correlation was performed between all these variables to ensure no redundancy between variables; correlation coefficients were always below |0.8|, the strongest correlation was between urbanization and the percentage of forested habitat (R = -0.65, p = 0.001, Table S2).

**References**

1.	Schuler, M. S. *et al.* Regulations are needed to protect freshwater ecosystems from salinization. *Philosophical Transactions of the Royal Society B: Biological Sciences* **374**, 20180019 (2018).

2.	Spyra, A., Cieplok, A. & Kaszyca-Taszakowska, N. From extremely acidic to alkaline: Aquatic invertebrates in forest mining lakes under the pressure of acidification. *International Review of Hydrobiology* **108**, 5–16 (2023).

3.	Mironov, D. Parameterization of lakes in numerical weather prediction. Part 1: Description of a lake model. Offenbach: Consortium for Small-scale Modeling. *Technical Report* **11**, 47 (2008).

4.	EEA. European Union, Copernicus Land Monitoring Service. *Eur. Environ. Agency* (2020).

5.	QGIS Development Team. QGIS geographic information system. Open source geospatial foundation project. (2017).

6.	Taylor, P. D. & Merriam, G. Wing morphology of a forest damselfly is related to landscape structure. *Oikos* 43–48 (1995).

**Table S2.** Spearman correlation analysis between urbanization (percentage of impervious surface), percentage of cropland and of forested habitat, water temperature for April, May and June, pH and salinity across the 22 ponds. Pearson correlation analysis between the three morphological traits measured on adults, hindwing size and forewing size (*N* = 796), males and females were analysed together. P-values were corrected for multitesting using Bonferroni correction (α/8 for the environmental parameters and α/5 for the morphological traits) and significant correlations are indicated in bold: ** p < 0.00125, * p < 0.00625, for the environmental parameters and *** p < 0.0002 for the traits measured on adults.

|  | Urbanization | pH | Salinity | Cropland | Forested | T_April | T_May |
| --- | --- | --- | --- | --- | --- | --- | --- |
| Urbanization | 1 |  |  |  |  |  |  |
| pH | -0.22 | 1 |  |  |  |  |  |
| Salinity | **0.62*** | -0.32 | 1 |  |  |  |  |
| Cropland | -0.14 | -0.28 | 0.09 | 1 |  |  |  |
| Forested | **-0.65**** | 0.19 | -0.32 | -0.25 | 1 |  |  |
| T_April | 0.19 | 0.04 | -0.04 | 0.17 | -0.13 | 1 |  |
| T_May | -0.13 | 0.05 | -0.18 | -0.03 | -0.15 | -0.45 | 1 |
| T_June | -0.1 | 0.37 | 0.01 | -0.02 | -0.1 | -0.1 | 0.49 |

|  | Hindwing size | Forewing size | Femur length | Thorax length |
| --- | --- | --- | --- | --- |
| Hindwing size | 1 |  |  |  |
| Forewing size | **0.94***** | 1 |  |  |
| Femur length | **0.39***** | **0.41***** | 1 |  |
| Thorax length | **0.70***** | **0.71***** | **0.4***** | **1** |
| Head width | **0.48***** | **0.49***** | **0.42***** | **0.58***** |

**Figure S1**. Enlarged map (© Stadia Maps © OpenMapTiles © OpenStreetMap; <https://stadiamaps.com/>, <https://openmaptiles.org/>, <https://www.openstreetmap.org/copyright>, R package ‘ggmap’ ^7^) showing the six sampled ponds within the city of Krakow.

**Reference**

7.	Kahle, D., & Wickham, H. (2013). ggmap: Spatial Visualization with ggplot2. version 4.0.2. <https://cran.r-project.org/web/packages/ggmap/index.html>, access 08.06.2026

**Figure S2**. Correlation between salinity and conductivity measured in 21 of the 22 ponds. For Schodno, values for conductivity were retrieved from previous research reports in 2019 and 2022 (https://www.gov.pl/web/gios/program-badan) and are equal to 721 and 731 µS.cm^-1^, respectively. We took the average across these two values (726 µS.cm^-1^) which corresponds to a value of salinity of 0.361 g.L^-1^.

**Figure S3**. Temperature measured with dataloggers placed at approx. 30 cm depth between 2021 and 2025. For two ponds: Schodno and Kryspinow, temperature was estimated using Flake ^3^. Temperatures from April to June, used in the analyses, was available for all ponds (some temperature values are missing for the other months). For some ponds, temperatures were recorded for several years between 2021 and 2025, in that case, we took the average across all years. Ponds are organized in alphabetical order and split into three panels for clarity.

**Fig. S4.** Allometry plot showing variation of the first principal component of the fitted values (predicted shapes) for the hindwings along the percentage of impervious surface. For females, shape deviation along the urbanization gradient is shown in the two panels below the plot. Grey dots represent the average position of each landmark across all females. Black arrows indicate deviation from the average configuration when the percentage of impervious surface is low (left panel) and high (right panel).

**Fig. S5.** Allometry plot showing variation of the first principal component of the fitted values (predicted shapes) for the forewings along the percentage of impervious surface. For females, shape deviation along the urbanization gradient is shown in the two panels below the plot. Grey dots represent the average position of each landmark across all females. Black arrows indicate deviation from the average configuration when the percentage of impervious surface is low (left panel) and high (right panel).

**Fig. S6.** Mean fluctuating asymmetry across the 22 ponds for a) the hindwings and b) the forewings. Large black dots represent the average position of each landmark between the right and left wing (perfect symmetry). Small grey dots represent deviation from the perfect symmetry for each individual.
